# Examination of Gender-Related Differential Item Functioning Through Poly-BW Indices

**DOI:** 10.3389/fpsyg.2022.821459

**Published:** 2022-02-25

**Authors:** Tsai-Wei Huang, Pei-Chen Wu, Magdalena Mo Ching Mok

**Affiliations:** ^1^Department of Counseling, National Chiayi University, Chiayi, Taiwan; ^2^Department of Educational Psychology and Counseling, National Pingtung University, Pingtung, Taiwan; ^3^Graduate Institute of Educational Information and Measurement, National Taichung University of Education, Taichung, Taiwan; ^4^Department of Psychology, Assessment Research Centre, The Education University of Hong Kong, Tai Po, Hong Kong SAR, China

**Keywords:** differential item functioning (DIF), Poly-SIBTEST, Poly-BW indices, teacher-made mathematics test, item-fit statistics

## Abstract

The existing differential item functioning (DIF) detection approaches relying on item difficulty or item discrimination are limited for understanding the associates of DIF items, and consequently, DIF items were conventionally either deleted or ignored. Given the importance of minimizing DIF items in test construction, teachers or testing practitioners need more information regarding possible associates of DIF items. Using an example of a teacher-made mathematics achievement test, this study aimed to examine how the Poly-BW indices (power, defenselessness, disturbance, and hint) contributed to the properties of gender-related DIF items. Data from a 34-item mathematics achievement test that involved 1,439 seventh-grade students from Taiwan (51.01% boys and 48.99% girls) showed that the differences of the defenselessness (mp) and power (cp) indices between men and women served as salient predictors of the DIF measures estimated by the Poly Simultaneous Item Bias Test (Poly-SIBTEST) procedure and with satisfactory accuracy of hit rates. Items with relatively large defenselessness for men were likely to present male-favoring DIFs, whereas items with relatively large power for men were likely to present female-favoring DIFs. The Poly-BW indices yielded directions for modifying items for teachers in practice.

## Introduction

Differential item functioning (DIF) indicates the situation where participants from different memberships (e.g., age and gender) on the same level of the latent trait (e.g., math performance) have a different probability of a certain response to a particular item (Holland and Thayer, 1988). DIF may hamper the interpretation of mean comparisons or even lead to misleading conclusions as an item with DIF yields either constant benefits for a particular membership (uniform DIF) or benefits differing in direction and magnitude across various memberships (non-uniform DIF).

Given the distorting consequences of DIF, various DIF techniques have been advanced to assess invariance in both person and item parameters. However, most DIF detection approaches and the estimation of DIF effect sizes are limited in providing the possible associates of DIF items (Kim et al., 2007; Li, 2014a,b, 2015). The possible associates of DIF-flagged items entail the possible reasons why the DIF occurs and through which we could determine whether these items should be deleted or revised. On the other hand, if the information on possible associates of DIF items is limited, the DIF items cannot be treated appropriately. Consequently, some researchers chose to remove a DIF-flagged item from the item bank, while others might conduct further analyses (e.g., Wang et al., 2012; Chen and Hwu, 2018). Furthermore, by reviewing 27 studies on DIF item treatment, Cho et al. (2016) found that 30% of the studies removed the DIF items while 26% of the studies ignored them.

The associates of DIF are complex and could be examined from several perspectives. Some studies developed multi-dimensional DIF detection methods to explain DIF resulting from the secondary dimension (e.g., Roussos and Stout, 1996; Mazor et al., 1998; Lee et al., 2016). Some studies explore differential distractor functioning (DDF) to express that DIF could be derived from distractors being attracted differently by individuals from different groups (e.g., Suh and Bolt, 2011; Suh and Talley, 2015) and others argued that the presence of DIF is not directly related to the defined groups but associated with a latent or unknown group (De Ayala et al., 2002; Cohen and Bolt, 2005). Item characteristics, person effects, and the interaction between item and person effects could be accounted for DIF. To provide implications for teacher-made tests in classrooms (e.g., modifications for DIF items based on properties of items), however, this study would investigate the associates of DIF with an emphasis on item effects (e.g., properties of items).

The properties of items delineate item characteristics (e.g., item difficulty), which could provide teachers or testing practitioners directions to modify or revise items exhibiting DIF. Two common properties are item difficulty and item discrimination. However, both of them are limited in addressing possible associates of DIF. Beyond that, aberrant response indices derived from a response pattern in an item could be alternative options to identify possible associates in DIFs. Due to the function of detecting the aberrant response patterns by assessing the agreement of difficulty within an item response pattern, the caution index (*SCI*, Sato, 1975) or modified caution index (*MCI*, Harnisch and Linn, 1981) developed by early researchers could be used to identify possible associates in DIF items. However, they are still limited by their ineffectiveness in distinguishing extreme levels of item difficulty (Huang and Wu, 2013). For example, a response pattern, such as 000| 00000111, consisting of “0” (representing a wrong response) and “1” (representing a correct response) is ranged by difficulty levels from left-easy items to right-hard items. Two different response patterns of the within-difficulty response pattern (000 before the symbol “|”) and the beyond-difficulty response pattern (111 after the symbol “|”) are handled by the *MCI* or *SCI* identically, even though these two response patterns might be due to different item characteristics. That is, possible intrinsic properties of item response patterns might not be distinguished by the *SCI* or by the *MCI* measures alone.

As an extension of the *SCI* measure, the beyond-surprise/within-concern dichotomous *BW* indicators (Huang, 2012; Huang and Wu, 2013) have yielded more useful information for the intrinsic properties of items. The dichotomous *BW* indicators provide the information of item characteristics about power (*c*), defenselessness (*m*), disturbance (*b*), and hint (*w*) for each item. The four indicators are useful for exploring the intrinsic properties of an item (Huang and Wu, 2013). Recently, the original dichotomous *BW* indicators have been extended to the *Poly-BW* indices for polytomously scored items (Huang and Lu, 2017), in which they are recorded as *cp, mp*, *bp*, and *wp*, respectively. However, no studies have yet examined the association between these four polytomous indices and DIFs. Furthermore, since the *Poly-BW* indices are non-parametric-based to yield diagnostic information for items (e.g., whether the items respond normally or aberrantly), they can provide information on item classifications by means of the approximate permutation test (APT, Edgington, 1995). Through the APT process, many data matrixes can be approximately simulated according to an original data matrix so that the 95th percentile values of individual indices in an item can be set and classified. This analysis enables us to disclose possible associates in suspective DIF items and by which DIF items could be more appropriately dealt with. Details of the item classification procedures are provided in the “Materials and Methods” section.

On the other hand, although recently, there are several existing DIF detection methods depending on different assumptions of their models, the Poly Simultaneous Item Bias Test (Poly-SIBTEST) approach is a wildly used non-parametric-based DIF-detecting procedure in polytomously scoring situations (Chang et al., 1996). Capitalizing on its non-parametric-based and polytomously scoring characteristics, the *Poly-SIBTEST* procedure was chosen in the current study as a reference method to estimate DIFs for examining the associations between the estimated DIF measures and the non-parametric-based *Poly-BW* indices measures.

Given the importance of the possible associates of DIF items, this study exemplifies the advantages of the four *Poly-BW* indices by assessing their predictive effects on the gender-related DIFs measured from the *Poly-SIBTEST* approach in a teacher-made math test. A gender-related DIF in a math text is a particularly important issue because gender difference in math performance has been debated for some decades. Some researchers argued that there were no gender differences in math performance (Hyde and Mertz, 2009; Lindberg et al., 2010), but others proposed that boys outperformed girls since third grade (Fryer and Levitt, 2010; Robinson and Theule Lubienski, 2011), and still other researchers showed that girls performed similarly or better than boys in terms of classroom grades (Pomerantz et al., 2002; Ding et al., 2006). However, these comparisons are valid only when a math test is established freely with the gender invariance. Accordingly, two main research questions guide this study: (1) Do the four *Poly-BW* indices predict effectively a DIF measure obtained from the *Poly-SIBTEST* approach? (2) How accurately do the four *Poly-BW* indices predict a DIF item?

### Dichotomous *BW* Indicators

Aberrant responses, known as inconsistent or unexpected responses compared to the overall responses in a test, can provide diagnostic information for persons and items (Kogut, 1986; Seol, 1998). Indices developed for detecting a person’s aberrant response patterns were usually labeled as person-fit indices, while for detecting an item’s aberrant responses was called item-fit indices. Some indices are based on the characteristics of a group, and others are based on item response theory (IRT) models (Harnisch and Linn, 1981; Kogut, 1986; Meijer and Sijtsma, 1999). Based on the group-based Guttman (1944) principles (1944) (i.e., able persons should answer those items correctly, which exhibited difficulty levels lower than those persons’ ability levels), the dichotomous *BW* indicators (Huang, 2002) were designed to detect persons’ aberrant responses patterns, i.e., person-fit oriented, and were subsequently extended to detect aberrant responses of items, i.e., item-fit oriented. The person-fit *BW* indicators originally (Huang, 2002) measured a person’s tendency of “*guess”* (*B* indicator) and “*carelessness”* (*W* indicator) and later (Huang and Wu, 2013) extended to measure a person’s tendency of “*mastery”* (*C* indicator) and “*misconception”* (*M* indicator), respectively. Huang (2011) investigated the robustness of *BW* aberrance indices against test length and found that the person-fit *BW* indicators were almost unrelated to test length. Later, Huang (2012) compared the aberrance detection powers among the person-fit BW indicators, other four group-based indicators (*SCI, MCI, NCI*, and *Wc & Bs*), and five IRT-based indicators (*OUTFITz, INFITz, ECI2z, ECI4z*, and *lz*) under the conditions of content category, type of aberrance, the severity of aberrance, and the ratios of aberrance persons. He found the person-fit BW indicators and the four group-based indices exhibited higher detection rates (over 90%) than the five IRT-based indicators, and furthermore, the BW indicators exhibited the best stability across different situations.

According to symmetrical characteristics, the properties of the item-fit *BW* indicators are the same as those of the person-fit *BW* indicators. Specifically, the dichotomous *BW* item-fit indicators (Huang, 2002) were developed to detect the tendency of “*disturbance”* (*b* indicator) and “*hint”* (*w* indicator) in an item beyond and within the item’s difficulty level, respectively. In addition to the two aberrant indicators, Huang and Wu (2013) incorporated another two indicators with normal responses for an item, called “*power*” (*c* indicator) and “*defenselessness*” (*m* indicator) within and beyond the item’s difficulty level, respectively. Here, normal responses mean those responses that obey the Guttman’s principles. In their study, a cognitive diagnostic model based on the *WBstar* program was used to examine the quality of a teacher-made test (22 items) on the contents of fractions and decimals and found that the *BW-based* cognitive diagnostic model performed effectively for detecting the misfit of items in small-sample scenario of 32 fourth-grade students.

For applying to a DIF scenario, Huang and Lin (2017) conducted a Monte Carlo study to investigate the predictive effects of the dichotomous *BW* indicators on DIFs. They simulated data under five conditions (sample size, item number, DIF type, DIF ratio, and DIF severity) and found that the power indicator and the defenselessness indicator could significantly explain the variances of DIFs. Moreover, the four dichotomous *BW* indicators also exhibited over 90% of the accuracy rate of prediction on the flagged-DIF items. However, Huang and Lin’s study was limited in the dichotomously scoring system and not suitably interpreted in polytomously scoring scenarios. In recognition of limited usage of the dichotomous *BW* indicators, Huang and Lu (2017) extended these indicators to fit in polytomously scored scenarios, called the *Poly-BW* indices. In their study, they developed the *PWBstar*1.0 program^1^ for estimating the *Poly-BW* indices, but they did not investigate the associations between the *Poly-BW* indices and DIF measures. Therefore, the predictive effectiveness of the *Poly-BW* indices to DIF measures is unknown.

### *Poly-BW* Indices

Similar to the dichotomous situations, the main idea of the *Poly-BW* indices was based on the concept of a discrepancy, which would reflect the level of aberrant or normal responses. The discrepancy distances between persons’ abilities and an item’s difficulty would be calculated according to normal or aberrant responses beyond and within an item’s difficulty level, respectively. Specifically, suppose in a *K*-item test participated by *N* examinees, we can first rank ability levels by individual person scores from the bottom (low) to top (high) and then rank difficulty levels by individual item scores from left (low) to right (high). Let the *j*th item has a maximum score of *s*_*j*_ and the *i*th person has an earned score *x*_*ij*_ on the *j*th item, then *x*_*ij*_ ≤ *s*_*j*_. Since the *N* examinees are ranked by individual’s total earned scores on the *K*-item test from bottom to top, we can define $t_{i}=\sum_{j=1}^{K}x_{ij}/\sum_{j=1}^{K}s_{j}$ as the *i*th person’s potential ability. Then we correspondingly calculate the accumulated maximum score by defining a specified *I*_*j*_ (1 ≤ *I*_*j*_ ≤ *N*) such that $I_{j}\times s_{j}\leq Q_{j}=\sum_{i=1}^{N}\left(s_{j}-x_{ij}\right)<\left(I_{j}+1\right)\times s_{j}$ for all *N* examinees from bottom to top, indicating a certain item score (*Q*_*j*_) defined as the difference between the maximum score and the earned score is falling between two sequential accumulated maximum scores and thus exhibits the item’s robustness so as for all *N* examinees not to answer it correctly. Ideally, the *i*th person who possesses the complete potential ability *t*_*i*_ should gain the accumulated maximum score on the *j*th item.

Therefore, the true item difficulty ($t_{Qj}^{*}$) for the *j*th item can be expressed by two sequential persons’ potential ability levels (*t*_*i*_ and *t*_*i+1*_) through interpolation technique with a 0.5 error unit as Equation 1 shows:


(1) $$t_{Q_{j}}^{*}=t_{i}+\left(\frac{Q_{j}-I_{j}\times s_{j}+0.5}{s_{j}}\right)\times \left(t_{i+1}-t_{i}\right)$$

Adding a 0.5 error unit in Equation 1 is because the score unit is 1 and using half of the score unit for correction will be helpful to distinguish difficulty levels when the examinee sample is too small.

With the true item difficulty ($t_{Qj}^{*}$) and based on the dichotomous logic, the *Poly-BW* indices (*cp, mp, bp*, and *wp*) designed to measure the degree of *power*, *defenselessness*, *disturbance*, and, *hint* for an item, respectively, are given from Equations 2 to 5 as follows:


(2) $$cp_{j}=\frac{\sum_{i=1}^{I_{j}}\left(1-\frac{x_{ij}}{s_{j}}\right)\times \left(t_{Q_{j}}^{*}-t_{i}\right)}{\left[\left(N-1\right)/2\right]}$$

(3) $$mp_{j}=\frac{\sum_{i=I_{j}+1}^{N}\left(\frac{x_{ij}}{s_{j}}\right)\times \left(t_{i}-t_{Q_{j}}^{*}\right)}{\left[\left(N-1\right)/2\right]}$$

(4) $$bp_{j}=\frac{\sum_{i=I_{j}+1}^{N}\left(1-\frac{x_{ij}}{s_{j}}\right)\times \left(t_{i}-t_{Q_{j}}^{*}\right)}{\left[\left(N-1\right)/2\right]}$$

(5) $$wp_{j}=\frac{\sum_{i=1}^{I_{j}}\left(\frac{x_{ij}}{s_{j}}\right)\times \left(t_{Q_{j}}^{*}-t_{i}\right)}{\left[\left(N-1\right)/2\right]}$$

The properties of the four *Poly-BW* indices are delineated as follows. First, the *power index* (*cp*) refers to the power of an item, that is, the examinees would answer it incorrectly just truly due to their ability is lower than the item difficulty (i.e., within the difficulty level). Second, the *defenselessness index* (*mp*) refers to the possible inefficiencies of an item, that is, examinees whose ability levels were higher than the difficulty level of the item (i.e., beyond the difficulty level) would easily answer this item correctly. The *cp* and *mp* indices differ from the difficulty index in the classical test theory (CTT). In CTT, the computation of the difficulty index does not distinguish “within” or “beyond” an item difficulty level. In this study, the *cp* index measures the part of “not correct” responses within the item difficulty, and the *mp* index measures the part of “correct” responses beyond the item difficulty. An item with a greater value of *power index* is more likely to be answered “incorrectly” than another item with a lower value of *power index*. Therefore, when comparing *power index* between two groups (gender groups), a greater value of *power index* for a particular item in the focal group (e.g., men) indicates that this item is more likely to be answered incorrectly by male examinees than by female examinees, given those examinees’ ability within this item difficulty. In contrast, an item with a greater value of *defenselessness index* is more likely to be answered “correctly” than another item with a lower value of *defenselessness index*. Therefore, when comparing the *defenselessness index* between two groups (gender groups), a greater value of *defenselessness index* for a particular item in the focal group (e.g., men) means that this item is more likely to be answered correctly by male examinees than by female examinees, given those examinees’ ability beyond this item difficulty.

On the other hand, the *bp* and *wp* indices indicate that an item displays possible aberrances of *disturbances* or *hints* that examinees might encounter, respectively. The *disturbance index* (*bp*) measures the property of an item with possible pitfalls, that is, examinees answer the item incorrectly even though their ability levels are beyond the difficulty level of the item. An item with a greater value of *disturbance index* is more likely to be answered “incorrectly” than another item with a lower value of *disturbance index*. Therefore, when comparing the *disturbance index* between two groups (gender groups), a greater value of *disturbance index* for a particular item in the focal group (e.g., men) suggests that this item has more likelihood to be answered wrongly for male examinees than female examinees, even given those examinees’ abilities beyond the item difficulty. In contrast, the *hint index* (*wp*) measures the possible prompts in an item, that is, examinees answer the item correctly even though their ability levels are within the difficulty level of this item. An item with a greater value of *hint index* is more likely to be answered “correctly” than another item with a lower value of *hint index*. Thus, a greater value of *hint index* for a particular item in the focal group (e.g., men) delineates that this item has more likelihood to be answered “correctly” for male examinees than female examinees, even given those examinees’ abilities are within the item difficulty.

**FIGURE 1:**
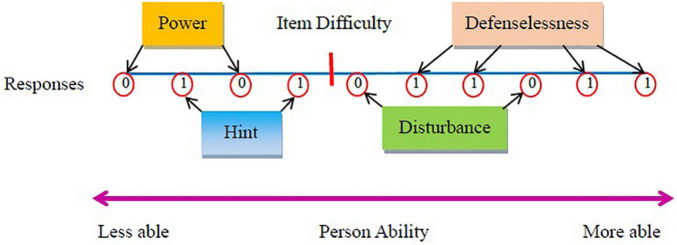
Ideas of the four *Poly-BW* indices beyond and within item difficulty.

Figure 1 provides a visualization for the idea of the four *Poly-BW* indices in different situations of normal and aberrant responses. Ideally, an item is supposed to be answered incorrectly when examinees’ abilities are lower than the item difficulty (i.e., *power*) and to be answered correctly when examinees’ abilities are higher than the item difficulty (i.e., *defenselessness*). On the other hand, when the ideal principles of responses are violated, the levels of discrepancy will reflect the levels of aberrance. The *disturbance* situation occurs when an item is still answered incorrectly even though examinees’ abilities are higher than the item difficulty. The *hint* situation occurs when an item is still answered correctly even though examinees’ abilities are lower than the item difficulty.

## Materials and Methods

### Participants

A total of 1,439 seventh-grade students from central Taiwan (51.01% men and 48.99% women) participated in the study. They were selected from 40 classes in eight junior high schools. The students completed a math test in a math class within around 40 min. In Taiwan, all math textbooks are developed based on directions governing the *Basic Education Curricula* and the contents of math textbooks should be approved by the Ministry of Education. Therefore, all junior-high-school students with the same grade are taught similar contents or units of math. Before administering this math test, all participants were supposed to have learned the contents of arithmetic, algebra, and geometry during their math class. We confirmed that all teachers had taught these contents before giving this math test.

### Instrument

The math test was developed by our research team, i.e., three junior-high-school teachers and two experts in math and psychological measurement. It was a criterion-referenced and formative assessment. The objective of this test was to evaluate what the students had learned and assessed whether students obtained the required knowledge. Therefore, all items were developed according to the directions governing the *Basic Education Curricula* in Taiwan. The test comprised 34 items involving the three major components of math: 14 items for *arithmetic* (such as addition/subtraction of integers, multiplication/division of integers, operations on fractions, and the concept of factors/multiples), 16 items for *algebra* (such as the operation/application of integers and fractions), and 4 items for *geometry* (such as the concepts of the number line and absolute numbers). Each item required more than two problem-solving steps to solve and was scored on the basis of the number of steps taken by the students (according to the rubric of the math test). Supplementary Appendix A presents the specifications of items by contents and formats. Supplementary Appendix B presents the original teacher-made math test.

### Rubric of Test

The rubric was established on the basis of the rationale of the partial credit model (PCM) (Masters, 1982). According to the PCM, to proceed with the next problem-solving step, the previous step is required to be correct. For instance, in the case of a three-step item, if a student answers the first step correctly but fails in the second step, it is reasonable to assume that he/she cannot answer correctly in the third step. Thus, based on the requirement of the PCM, each correct step, given that the previous steps were correct, was given one point, but a step was not given a point if any previous step was incorrect. Thus, the possible scores for each item in this study, ranging from 0 to 2, 0 to 3, 0 to 4, and 0 to 5, depended on the total number of solution steps and the corresponding previous correct steps for each item. To validate the step scores, two raters were asked to judge the step scores and to add the total scores for all the examinees on each item. The Pearson product-moment correlations were calculated between the two columns of total scores from the two raters for all the examinees on each item and ranged from 0.88 to 0.99, indicating good levels of inter-rater consistency for all items. All intracorrelations between items were significant at 0.01 alpha level and shown in Supplementary Appendix C by the male and female groups, respectively.

### *Poly-SIBTEST* Procedure

The *Poly-SIBTEST*, a confirmatory and theory-driven approach to detect associates of DIF, was designed to address the problem of DIF identification in polytomously scoring situations (Chang et al., 1996). *Poly-SIBTEST* is a non-parametric method (i.e., it did not assume the parametric shape of the item response function) and it was a Shealy-Stout multidimensional model (MDM; Shealy and Stout, 1993) used for DIF detection. The MDM model delineates that a DIF is a result of the second construct that is not intended to measure in a test (Shealy and Stout, 1993). Put differently, a DIF item measures more than one construct and the focal group (studied group) have different scores in the second construct. Therefore, according to MDM, a DIF occurs when the reference and focal groups that are matched on the same level of the intended (main) construct have different scores (distributions) on the second construct.

Based on *Poly-SIBTEST* theory and prior research, when items are hypothesized to have a common secondary construct, they could be bundled together and assessed for differential bundle functioning (DBF). Specifically, the inequality *T*_*jF*_(θ) < *T*_*jR*_(θ), where θ represents the measured target ability, *T* represents the marginal item response functions on item *j* for the focal group (*F*) and the reference group (*R*), respectively. The difference between the subtest response functions gives a preliminary index of DBF, given the examinees’ ability level. If all ability levels are considered, an index of DBF, i.e., *Bu*, can be estimated. The estimate of *Bu* can be tested by the standardized statistic, which has an approximately normal distribution with a mean 0 and SD of 1 for a large sample. Items with values greater than 1.96 or less than − 1.96 are deemed as DIF items. In *Poly-SIBTEST* analysis, we set the female group as the reference group so that the values of *Bu* greater than 0 indicate male-favoring potential and values of *Bu* less than 0 indicate female-favoring potential. However, for a DIF-flagged item, only the values of the standardized *Bu* greater than 1.96 or less than − 1.96 were considered.

### Valid Matching Subtest

Before DIF estimation, a valid matching subset was required previously in the *Poly-SIBTEST* procedure. There were 18 of 34 items of interest, such as mathematics content (arithmetic, algebra, and geometry), number type (fraction and integer), and item format (operation and word problem), which were classified into five bundles of the studied subtest. The remaining 16 items, after precluding the items in the studied subtest, were used in the matching subtest for the purpose of purification in the first automatic DIF analysis (Stout and Roussos, 1995). After conducting automatic DIF analysis three times and canceling five items that displayed significant DIFs, a valid matching subtest consisting of 11 non-DIF items was found (item 1, 6, 8, 10, 12, 13, 15, 20, 22, 27, and 32). These uncontaminated items were used to identify the ability levels of male and female students in *Poly-SIBTEST* analysis.

### Analysis

In this study, we set women as the referenced group in both *Poly-SIBTEST* analysis and *Poly-BW* differences estimation. A positive DIF measure in *Poly-SIBTEST* analysis indicated a male-favoring item and a negative DIF measure indicated a female-favoring item. A positive index difference in *Poly-BW* analysis implied a certain *Poly-BW* index estimated from the male group greater than that estimated from the female group, and vice versa. Thus, for research question 1, the *Poly-BW* indices were calculated for the male and female groups separately through the *PWBstar*1.0 program, and the differences in individual indices between the two groups were used as predictors for the DIF measure (*Bu*) obtained from *Poly-SIBTEST* through stepwise multiple regression analysis. For research question 2, the accuracy of classification for a DIF-flagged item by the four *Poly-BW* indices from the APT procedure was assessed by multiple discriminant analysis.

Specifically, the study used a 100-repetition APT procedure through the following steps: (1) setting three types of *cp* values (high, middle, and low, using 33% ranks as cutoffs); (2) setting two types of levels (high and low) by using upper or lower the 95% percentile value for each *mp*, *bp*, and *wp* index in the male and female groups, respectively. After these APT steps, all information on the item classifications can be provided by the *PWBstar* 1.0 program by labeling the types of item classifications as (letter + number)’, in which the letters represent high (H), middle (M), and low (L) *power* levels of the *cp* index; while the numbers 1, 2, 3, and 4 refer to *normal*, *disturbance*, *hint*, and *hybrid* (disturbance plus hint) types of responses, respectively. Further, the prime symbol (′) indicates a significantly high *defenselessness* value of the *mp* index. The item classifications provide insights into the associates of DIF. For example, if a DIF item is labeled as L1′ and L2′ for men and women, respectively, it implies that this DIF item displays a low level of *power* (L) and a high level of *defenselessness* (′) for both genders, but with *normal* performances (“1”) for the male groups and aberrant *disturbances* (“2”) for women. Suspiciously, disturbance might be an associate of DIF.

## Results

### Descriptive Statistics and Preliminary Analysis of Dimensionality

Descriptive statistics of item level performance and parameters estimated by the *Poly-WB* item indices formula for men (*N* = 734) and women (*N* = 705) were displayed in Supplementary Appendix D. Due to different problem-solving steps required in individual items, the scores of items were ranged from 0 to 2, 0 to 3, 0 to 4, and 0 to 5. As can be seen in Supplementary Appendix D, the mean scores on an item earned by male students ranged from 0.54 (max score 3 in item 29) to 3.48 (max score 5 in item 26) and ranged from 0.78 (max score 2 in item 30) to 3.85 (max score 5 in item 26) by female students. The smallest SDs for both groups occurred on item 19 (0.81 and 0.74 for men and women, respectively) and the largest SDs occurred on item 23 (2.34 and 2.42 for men and women, respectively). Before examining DIFs, the dimensionality of these 34 items was preliminarily checked. Principal components analysis of standardized residuals showed that unexplained variance explained by the main dimension was only 0.8%, indicating this math test exhibited a unidimensionality.

### Prediction

Table 1 summarizes the stepwise multiple regression analysis. Two *Poly-BW* indices explain almost 78% of the variance (63.8% from the predictor of *mp*_*M–F*_ and 14.2% from the predictor of *cp*_*M–F*_) of the DIF measures (*Bu*) obtained from *Poly-SIBTEST* analysis (*F*_4,29_ = 28.53, *p* < 0.001). Specifically, the *defenselessness (mp) index* has the largest contribution (63.8%), followed by the *power (cp) index* (14.2%). This finding indicates that the *defenselessness* (*mp*) and *power (cp) indices* could be used to predict the gender DIF measures in the math test (β = 0.734, *p* < 0.001 for *mp*_*M–F*_; β = − 0.286, *p* < 0.05 for *cp*_*M–F*_)^2^. Because the women were regarded as the referenced group in both *Poly-SIBTEST* analysis and *Poly-BW* difference estimations, a positive regression coefficient implied that the larger the *defenselessness* differences (*mp*_*M–F*_), the larger are the DIF measures (i.e., the items were more likely to favor men when they had a relatively large defenselessness for men). Likewise, a negative regression coefficient indicated that the larger the *power* differences, the smaller are the DIF measures (i.e., the items were more likely to favor women when they had a relatively large power for men). In summary, the *defenselessness* (*mp*) and *power (cp) indices* were significant predictors of DIF properties in this case. From the definitions of these two indices, these findings indicated that if an item was perceived as more defenseless (easy to be answered correctly beyond the item’s difficulty) or powerful (hard to be answered correctly within the item’s difficulty) by one gender group, then the item was more likely to exhibit a DIF. Interestingly, the abnormal indices (*hint wp* and *disturbance bp*) did not contribute significantly to the DIF properties. This finding indicated that these two abnormal indices were less likely to be the possible associates of DIFs in this case.

**TABLE 1:** Regression model summary for *Poly-BW* indices on differential item functioning (DIF) measures.

		*SS*	*df*	*MS*	*F*	*p*	*R* ^2^		
*ANOVA*	Regression	0.300	4	0.075	28.53	<0.001	0.797		
	Residual	0.076	29	0.003					
	Total	0.376	33						

		**Var.**	***R***	**Δ*R^2^***	**λ**	**S.E.**	***B***	***t***	***p***

Coefficients		Con.			0.082	0.015		5.358	<0.001
		*mp*_*M–F*_	0.799	0.638	1.952	0.298	0.734	6.543	<0.001
		*cp*_*M–F*_	0.883	0.142	−0.686	0.300	−0.286	−2.285	0.030
		*wp*_*M–F*_	0.892	0.015	2.970	2.437	0.138	1.219	0.233
		*bp*_*M–F*_	0.893	0.002	1.441	2.401	0.071	0.600	0.553

*Dependent Variable: Bu.*

*Subscripts in selected variables (e.g., mp_M–F_) represent the difference in an index value between male and female.*

Furthermore, to demonstrate the *Poly-BW* indices outperformed the difficulty index of CTT, i.e., *P* = (*P*_*H*_ + *P*_*L*_)/2 in terms of DIF variance explained, we compared DIF variance explained from the *P* index and the *Poly-BW* indices. Results showed that the CTT difficulty index explained 53.4% variance of DIF measure*s* (*F*_1,32_ = 36.68, *p* < 0.001), but it was lower than the variance explained by the *defenselessness* (*mp*) *index* (63.8%). Since the difficulty index based on CTT is calculated based on total scores, it confounded the within-difficulty and the beyond-difficulty effect in a person’s responses. Instead, the within-difficulty and beyond-difficulty effects can be distinguished by the *power (cp) index* and the *defenselessness* (*mp*) *index*, respectively, so that the intrinsic properties in DIFs can be more disclosed.

### Accuracy

**FIGURE 2:**
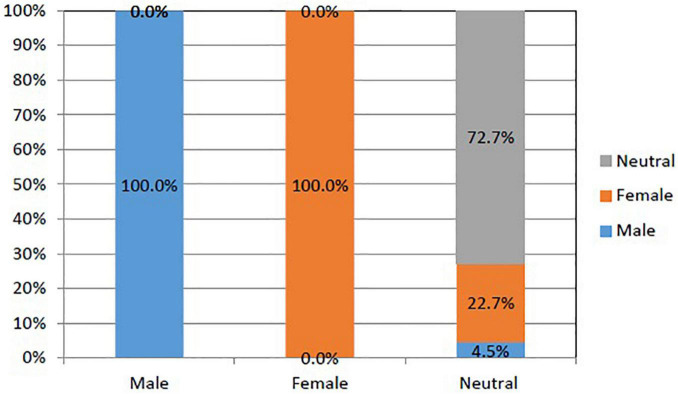
Hit rates of differential item functioning (DIF)-flagged items according to multiple discriminant analysis.

For research question 2, we examined how accurately the four *Poly-BW* indices predicted the DIF-flagged items using multiple discriminant analysis. Based on the standardized values of *Bu* obtained from the *Poly-SIBTEST* procedure (i.e.,|*Bu*| > 1.96), 12 items were detected with DIFs: 6 items favoring men (items 2, 5, 7, 11, 14, and 17) and 6 items favoring women (items 4, 18, 19, 21, 29, and 31). The remaining 22 items were neutral to both genders. The average hit rate of the DIFs (see Figure 2) was 82.4%. Without considering the group favored by the DIF items, the DIFs were perfectly (100%) predicted by the four *Poly-BW* indices. But the neutral category was predicted with an accuracy of 72.7% with six undetermined items. There were five neutral items (items 22, 23, 26, 27, and 28) classified as female-favoring items and one neutral item (item 25) classified as a male-favoring item.

When examining the values of *Poly-BW* indices (see Table 2), we found that five of six male-favoring items (except item 7) have higher *mp* values in the male group than in the female group, and all six female-favoring items have higher *mp* values in the female group than in the male group. For the undetermined items, the six neutral items displayed higher *cp* values in the male group than in the female group, but different magnitudes of *mp* values in both groups. All the five female-favoring items displayed higher *mp* values in the female group than in the male group, but in contrast, the male-favoring item (item 25) displayed a higher *mp* value in the male group than in the female group (0.094 vs. 0.076). This implies that the *mp* index (*defenselessness*) dominates the key reasons of item transformation from no DIF to favoring one group. Other *Poly-BW* indices values of remaining items can be seen in Supplementary Appendix D.

**TABLE 2:** Values of *Poly-BW* indices for differential item functioning (DIF) items and undetermined items.

		Raw Mean		Raw SD		*cp*		*mp*		*bp*		*wp*	
Category	Item	Male	Female	Male	Female	Male	Female	Male	Female	Male	Female	Male	Female
Male-favoring	v2	1.30	1.26	0.94	0.95	0.084	0.117	0.416	0.352	0.056	0.047	0.026	0.026
	v5	1.27	1.29	0.95	0.94	0.099	0.113	0.420	0.385	0.032	0.029	0.022	0.021
	v7	1.65	1.76	1.38	1.34	0.164	0.150	0.321	0.326	0.036	0.027	0.025	0.022
	v11	1.23	1.23	0.97	0.96	0.114	0.130	0.398	0.351	0.028	0.025	0.024	0.028
	v14	1.84	1.86	1.87	1.85	0.263	0.256	0.215	0.197	0.034	0.029	0.029	0.030
	v17	1.03	1.04	1.64	1.61	0.583	0.559	0.062	0.048	0.012	0.009	0.017	0.022
Female- favoring	v4	1.68	1.99	1.24	1.16	0.141	0.080	0.308	0.375	0.065	0.072	0.035	0.037
	v18	1.34	1.52	0.92	0.82	0.080	0.041	0.463	0.576	0.034	0.030	0.017	0.013
	v19	1.32	1.50	0.81	0.74	0.081	0.048	0.429	0.520	0.056	0.045	0.022	0.020
	v21	0.95	1.18	1.33	1.39	0.478	0.359	0.099	0.133	0.017	0.020	0.018	0.024
	v29	0.54	0.80	1.10	1.26	0.710	0.521	0.023	0.040	0.009	0.022	0.034	0.046
	v31	1.40	1.68	1.43	1.39	0.267	0.174	0.217	0.291	0.032	0.036	0.024	0.018
Undetermined	v25	0.93	0.94	1.38	1.36	0.484	0.462	0.094	0.076	0.016	0.016	0.025	0.032
	v26	3.48	3.85	2.04	1.78	0.060	0.034	0.497	0.582	0.039	0.038	0.019	0.015
	v27	1.22	1.45	1.40	1.43	0.356	0.242	0.151	0.217	0.026	0.027	0.029	0.025
	v28	1.46	1.68	1.44	1.42	0.244	0.162	0.234	0.286	0.032	0.037	0.029	0.033
	v22	1.62	1.90	2.20	2.26	0.465	0.368	0.098	0.117	0.019	0.023	0.029	0.036
	v23	1.94	2.28	2.34	2.42	0.375	0.276	0.151	0.199	0.017	0.018	0.023	0.020

**FIGURE 3:**
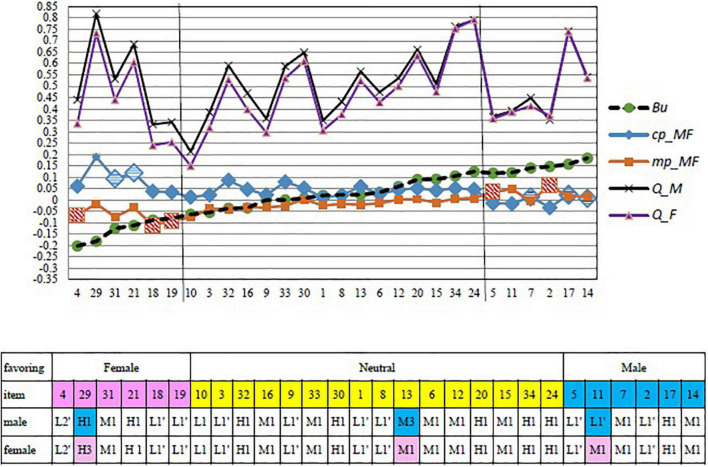
Differential item functioning (DIF) predicted by *mp* and *cp* indices and classifications for favoring items.

Furthermore, after excluding the six undetermined items, the remaining 28 items predicted by the *defenselessness* (*mp*) and *power* (*cp*) *indices* as male-favoring, female-favoring, and neutral are shown in Figure 3. As can be seen, in terms of the *Q* values from CTT, men mostly perceived an item as more difficult than women across all items, suggesting that CTT item difficulty cannot explain the possible associates of gender DIFs. Nevertheless, the *mp* and *cp* indices provide useful information on the associates of a DIF. First, in the female-favoring part (the left part of Figure 3), most of the female-favoring items exhibited low values of *defenselessness* (*mp*) but high values of *power* (*cp*) between men and women. Here, the values of *defenselessness* (*mp*) and *power* (*cp*) were the difference values between the gender groups (where women were the referenced group). Accordingly, when the items had lower *defenselessness* and higher *power* for men, they were likely to be female-favoring items. Second, in the male-favoring part (the right part of Figure 3), most of the male-favoring items exhibited high values of *defenselessness* (*mp*) but low values of power (*cp*) between men and women. Thus, when the items had higher *defenselessness* and lower *power* for men, they were likely to be male-favoring items.

Furthermore, the item classifications from the APT yielded more insightful information for DIF item treatment. In the female-favoring part, three items (i.e., items 4, 18, and 19) with high values of *defenselessness* (*mp*) exhibited consistent item types (L2′, L1′, and L1′, respectively) for both men and women. This implied that men and women consistently perceived the three items as defenseless. Moreover, they consistently perceived item 4 as defenseless and with disturbance (L2′), and items 18 and 19 as normal (L1′). Two other DIF items (items 31 and 21) with relatively high values of *power* (*cp*) exhibited consistent item types (M1 and H1, respectively) for both men and women, indicating that the two groups perceived the two items as having middle to high levels of power, respectively. On the other hand, only the item classification of item 29 was different for the gender groups: H3 for women and H1 for men. Thus, item 29 was perceived as having a high level of power for both groups but with a *hint* only for the female group. The *Hint* was likely an associate of this DIF item (item 29).

In the male-favoring part, six items exhibited male-favoring properties. Two of them (items 5 and 2) exhibited the same *defenselessness* item types (L1′) and three of them (items 7, 17, and 14) exhibited the same *power* item types (M1, H1, and, M1, respectively) for both groups. Thus, these findings implied that men and women consistently perceived the former two items as defenseless but the latter three items as having middle to have high levels of power. Interestingly, only item 11 was labeled as L1′ and M1 for men and women, respectively, suggesting that men perceived this item as more defenseless than women did, which likely resulted in its male-favoring property. In the neutral part, all 15 of 16 items performed consistently across the male and female groups. Only item 13 was labeled as M3 and M1 for men and women, respectively, indicating the item performed middle power for both groups, but men perceived more hints than women. Interestingly, item 13 did not perform DIF. This might be due to middle power performed by the item and, according to Table 1, the variances of DIFs explained by the power (*cp*) index are low (only 14.2%).

In summary, a 100-repetition APT found only two items (items 29 and 11) to be perceived with different item classifications for men and women among the 12 DIF items.

## Discussion

Given that the possible associates of DIF items have not attracted much attention, this study employed the *Poly-SIBTEST* approach as a reference method to demonstrate how the *Poly-BW* indices contribute to DIF measures with an example of the math test. To our best knowledge, this study may be the first study to investigate how the *Poly-BW* indices explain the possible associates of DIFs. There are several significant findings which were reported. First, two of the *Poly-BW* indices (*defenselessness* and *power*) significantly contributed to the *Poly-SIBTEST*-based DIF measures. This finding was largely consistent with the Monte Carlo study of Huang and Lin (2017), in which they simulated dichotomous data under five conditions (sample size, item number, DIF type, DIF ratio, and DIF severity) for Rasch-based DIF measures. In their study, they found that the *power index* and *defenselessness index* could on average predict Rasch-based DIFs with significant absolute beta values as 0.41 and 0.26, respectively, and explain 22.4% of total variances of DIFs.

In our polytomously-scoring study, the *defenselessness index* (*mp*) explained most of the variance of the DIF measures in this case, indicating that the extent to which an item is more likely to be answered correctly by the examinees whose ability levels were higher than the difficulty level of the item in one group than those in the other group is a significant predictor of the occurrence of DIF. More specifically, if an item is perceived as weak and exhibits high *defenselessness* so that persons with ability levels higher than the difficulty level are very easy to answer it correctly, then the item is likely to be identified as a DIF item. In addition and importantly, when compared with the difficulty index based on CTT, *defenselessness index* (*mp*) explained more variance of DIF measures because the *Poly-BW* index could clearly distinguish “within” or “beyond” the item difficulty level. If we ignore the within-beyond effect on DIF associates and just wholly deal with traditional difficulty index as an indicator of DIFs, then we may lose some useful information of possible associates in a DIF item. This is because the effects of *power* and *defenselessness* may be washed out in a single item.

Second, the *Poly-BW* indices provide clearer clues for understanding the different associates of DIFs for men and women in the math test. In this case, if an item has relatively high *defenselessness* and relatively low *power* for men, it is likely to be a male-favoring DIF item. By contrast, if an item has relatively low *defenselessness* and relatively high *power* for women, it is likely to be a female-favoring DIF item. The gaps of *defenselessness* or *power* between genders may be the possible reasons for gender-related DIF in the math test. Given the gap of *defenselessness* explaining the large DIF variance (63.8%), this study revealed that DIF mainly occurs in a certain situation where a person ability is beyond item difficulty. More specifically, the more difference on *defenselessness* exists between both genders, the more likely DIF occurs. In line with this finding, we suggest that the treatment of DIF items should depend on the type of assessment. If the assessment is a norm-referenced test, the DIF items with high *defenselessness* for both genders may be modified. Such an item should be modified as a more difficult item. By contrast, if the assessment is a criterion-referenced test, the DIF items with high *defenselessness* for both genders may be retained. Because the objective of criterion-referenced tests is simply to inspect whether the students have learned the materials, the items (relatively defenseless items) measuring the basic concept of the materials are commonly or necessarily included in the test.

Third, although we found the *Poly-BW* indices can precisely classify most of the DIF items identified by Poly-SIBTEST procedures, there were six neutral items misclassified as the female-favoring or male-favoring items. With more specific inspections, the *mp* index (*defenselessness*) dominates the key associates of DIF items and major transformation reasons from no DIF to favoring one group. The possible main reason for this discrepancy might be due to the Poly-SIBTEST procedures assessing a DIF based on the concept of a “whole” item score; however, the *Poly-BW* indices distinguish two response patterns (within-difficulty response pattern and the beyond-difficulty response pattern).

The findings in this study have some important implications. Teachers or testing practitioners could modify or revise DIF items based on the *Poly-BW* indices. When an item is flagged as DIF in practice, the possible reasons of DIF items, such as *defenselessness* or *power*, should be examined through the *Poly-BW* indices. From the *Poly-BW* indices, researchers and practitioners can understand possible associates of DIF items on math performance. Further treatment of the DIF items depends on the item classifications and the types of assessment. In addition, although the *disturbance* (*bp*) and *hint* (*wp*) are not significant predictors of DIF measures in this study, it does not mean that *disturbance* or *hint* is not the associates of DIFs in this case. It is likely that the effects of *disturbance* and *hint* on the DIF measures of all items are offset and negligible, but they may be significant for some items. Finally, item classifications involving complicated procedures in APTs could be easily conducted using the *PWBstar*1.0 program; thus, practitioners or teachers could employ this program to examine the intrinsic properties of tests.

This study has the following limitations. First, this study is the first study to explore how the *Poly-BW* indices contribute to DIF measures in a teacher-made mathematics achievement test; thus, the findings are more exploratory in the context of teacher-made math tests, and additional evidence is required for similar or different fields. For example, additional evidence as to whether the *defenselessness* property is the primary associate for DIF items in criterion-referenced tests should be obtained. Second, the significant predictors (*Poly-BW* indices) for DIF measures in norm-referenced tests still need to be explored. Third, this study used the Poly-SIBTEST index as a reference method, limiting to examine uniform DIF. The Crossing SIBTEST (e.g., Li, 2020) in assessing non-uniform DIF could be included in further studies. Finally, the *Poly-BW* indices include four indices for both persons and items. This study emphasizes the use of the *Poly-BW* indices for items; thus, future studies could investigate examinees’ performance using the *Poly-BW* person-fit indices.

## Data Availability Statement

The raw data supporting the conclusions of this article will be made available by the authors, without undue reservation.

## Ethics Statement

Ethical review and approval was not required for the study on human participants in accordance with the local legislation and institutional requirements. All participants voluntarily joined this study. The procedures of this study follow ethics principles for human research.

## Author Contributions

T-WH contributed for creating ideas, data collection, data analysis, and writing. P-CW contributed to data analysis, discussion of ideas, and revision of the manuscript. MM yielded feedback and critically revised the article for important intellectual content. All authors contributed to the interpretation of the results and read and approved the submitted version.

## Conflict of Interest

The authors declare that the research was conducted in the absence of any commercial or financial relationships that could be construed as a potential conflict of interest.

## Publisher’s Note

All claims expressed in this article are solely those of the authors and do not necessarily represent those of their affiliated organizations, or those of the publisher, the editors and the reviewers. Any product that may be evaluated in this article, or claim that may be made by its manufacturer, is not guaranteed or endorsed by the publisher.

## Footnotes

1
https://www.ncyu.edu.tw/gcweb/content.aspx?site_content_sn=44700

2 We also chose a 1-parameter Rasch model to estimate the DIF contrasts (men minus women). The results showed that three *Poly-BW* indices explain almost 88% of the variance (70% from the predictor of *mp*_*M–F*,_ 15% from the predictor of *cp*_*M–F*_, and 3% from the predictor of *w*_*M–F*_) of the DIF contrast obtained from Rasch DIF analysis. The findings were similar to those from the *Poly-SIBTEST* analysis. Because Rasch DIF analysis is not a non-parametric-based method, the results for further analyses were not conducted.
