# Supplementary material for: Examination of Gender-Related Differential Item Functioning Through Poly-BW Indices
Source: Front Psychol. 2022 Feb 25;13:821459. doi: 10.3389/fpsyg.2022.821459 (PMC8914429; doi:10.3389/fpsyg.2022.821459)
Supplement: Supplementary file 1 [file Data_Sheet_1.PDF]

Appendix A. Specifications of mathematics items by content and format

	Items (Max Scores)	Total score
	Item Content	
Arithmetic		41
Addition/Subtraction of Integer	2(2), 11(2), 19(2)	6
Multiplication/Division of Integer	1(2), 10(3), 27(3)	8
Operation of Fraction	3(3), 4(3), 28(3), 29(3)	12
Factor/Multiple	12(3), 13(4), 20(5), 21(3)	15
Algebra		54
Integer	6(2), 7(3), 8(3), 14(4), 18(2), 22(5), 23(5), 31(3), 32(4)	31
Fraction	9(3), 15(3), 16(3), 24(4), 25(3), 33(3), 34(4)	23
Geometry	5(2), 17(4), 26(5), 30(2)	13
	Item Format	
Operation/Computation	1, 2, 3, 4, 5, 6, 7, 8, 9, 27, 28, 29, 30, 31, 32, 33, 34	47
Word Problem		61
Consumption Problems	16, 23, 24, 25	15
Others	10, 11, 12, 13, 14, 15, 17, 18, 19, 20, 21, 22, 26	46
	**Total**	108

Appendix B. Math Test (For 7th grade middle school students)

1) Calculate $(-81)\div 3+(-27)\div(-9)$

2) Calculate $[(-34987695)+899931]+(-899930)$

3) Calculate $\frac{4}{3}-\frac{3}{4}+\left(-\frac{2}{5}\right)$

4) Calculate $4\frac{1}{5}\div 3\frac{2}{11}\times\left(-\frac{5}{9}\right)$

5) Calculate $|5-3|-|5-8|+|2-3|$

6) Simplify $5(-x+3)-3(2x-2)$

7) Solve $2(3-x)=-4(x+5)$

8) Solve $(4x-3)-(2x+1)=6$

9) Solve $\frac{2}{3}x+7=9$

10) A box of oranges weights 40 Taiwanese kilograms. Another box of apples weights 25 kilograms. Which box is heavier? By how much? (Note: 1 Taiwanese kilogram = 0.6 kilograms)

11) The lowest temperature in the world one day was –25℃. The same day the temperature in Taipei was 12℃. How much higher was the temperature in Taipei than the lowest temperature in the world on that day?

12) Which integers between 1 and 50 are still integers after they are multiplied by $\frac{1}{6}$ and $\frac{1}{8}$?

13) If a seven-digit number like 432□905 is a multiple of 3, what are the possible numbers of □?

14) Mr. Wang bought 30 kilograms of pears at the price of \$50 per kilogram. He selected 20 kilograms of good pears among the total, and sold them at the price of \$80 per kilogram. If he wants to earn 600 dollars in total after he has sold all the pears, how much per

kilogram should he label the bad pears?

15) The highest score in a math quiz among all the students is 100 points. What is John' actual score if one third of his score is equal to the highest score minus 20 points?

16) Ann's mother bought a skirt at 40% off. Ann checked the receipt and found the charged money was $234. How much was the original price for this skirt?

17) A, B, C, D, E are five points on the number line below. The distances between successive points are the same. If the position of point B is –2 and the position of point E is 5, what is the position of point A?

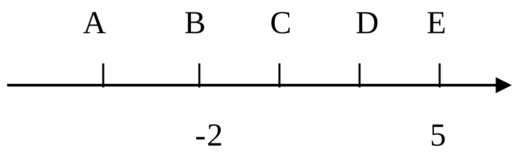

18) Tom has collected 90 baseball cards and David has collected 95 cards. If the number of baseball cards that Jeff has collected is more than Tom's but less than David's, what is the possible number of baseball cards that Jeff could have collected?

19) Peter managed a company. He lost $7850 in the first month. He earned $9870 in the second month, but lost $4320 in the third month. How much did he earn or lose during these three months?

20) Find the possible positive integer a such that $\frac{91}{a}$ is also a positive Integer (Multiple solutions).

21) If $a = 2^2 \times 5^3 \times 11^2 \times 13^2$ and b=2310, find the greatest common factor of both a and b. (The greatest common factor needs to be expressed in standard decomposition format, such as in a's expression)

22) The sum of five successive even numbers is 70. What are these five successive even numbers?

23) Tommy and his family bought movie tickets on Sunday. They spent $121 for two adult tickets and 3 child tickets. One adult ticket is $18 more expensive than one child ticket. How much is one adult ticket? One child ticket?

24)William used one third of his money to buy two books. He then used one half of the remaining money to buy 3 pencils. Afterwards, he had $200 left. How much money did William have originally?

25)A CD’s price was 20% off on Friday. Sandra used a 20-dollar coupon to buy the CD and paid $188 for it. What was the CD’s original price?

26）What are the numbers of A, B, C, D, and E on the number line below?

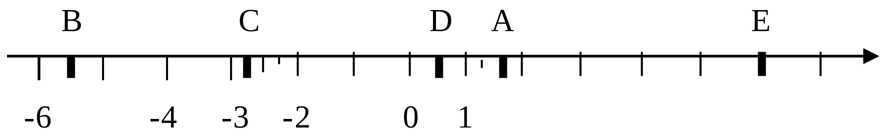

27) Calculate $23-2\times\left[5+\left(-2^3\right)\right]+7$

28) Calculate $\frac{74}{123}\times\frac{4}{5}+\frac{49}{123}\times\frac{4}{5}$

29) Calculate $4\div\left(-\frac{1}{2}\right)^3\times(-6)+\left(-3^2\right)$

30) Calculate $\frac{\left|3-\left|-5\right|\right|}{2}$

31) Solve $2(x-4)-(3x+4)=-20$

32) Solve $4\left[x-3(x-2)\right]+1=5$

33) Solve $\frac{1}{3}(x-3)=\frac{1}{4}(x+6)+1$

34) Solve $2x-\frac{x-5}{4}=17$

Appendix C. Item correlation by male and female groups

ITEM	v1	v2	v3	v4	v5	v6	v7	v8	v9	v10	v11	v12	v13	v14	v15	v16	v17	v18	v19	v20	v21	v22	v23	v24	v25	v26	v27	v28	v29	v30	v31	v32	v33	v34
v1	1	.39	.42	.42	.44	.40	.42	.48	.44	.38	.38	.40	.38	.35	.40	.35	.34	.38	.37	.36	.34	.32	.35	.29	.28	.40	.38	.38	.23	.32	.43	.41	.45	.32
v2	.42	1	.36	.40	.38	.37	.40	.42	.39	.34	.36	.43	.36	.38	.37	.40	.33	.38	.38	.39	.35	.30	.34	.28	.32	.37	.35	.38	.26	.34	.36	.37	.40	.31
v3	.38	.44	1	.49	.40	.39	.41	.44	.49	.40	.41	.40	.36	.41	.47	.39	.36	.42	.42	.44	.36	.35	.40	.32	.32	.39	.41	.41	.26	.34	.43	.34	.44	.30
v4	.35	.35	.42	1	.45	.47	.49	.49	.51	.41	.46	.48	.41	.45	.45	.42	.39	.42	.38	.46	.43	.37	.47	.33	.38	.35	.46	.45	.31	.39	.46	.43	.49	.37
v5	.42	.45	.42	.36	1	.49	.50	.54	.49	.45	.42	.48	.42	.44	.48	.44	.38	.44	.44	.45	.45	.43	.46	.33	.37	.42	.46	.46	.30	.42	.50	.48	.46	.36
v6	.43	.44	.44	.38	.50	1	.64	.66	.58	.38	.44	.48	.45	.40	.51	.44	.45	.41	.40	.43	.47	.42	.47	.32	.42	.38	.51	.45	.28	.40	.59	.60	.54	.43
v7	.44	.51	.41	.37	.49	.61	1	.68	.64	.42	.49	.53	.42	.43	.54	.44	.45	.40	.42	.48	.49	.45	.50	.40	.45	.43	.56	.47	.34	.44	.61	.62	.61	.45
v8	.49	.46	.43	.40	.51	.60	.69	1	.68	.43	.48	.50	.46	.43	.57	.42	.45	.38	.37	.49	.47	.44	.48	.39	.41	.40	.56	.47	.32	.46	.68	.63	.59	.43
v9	.51	.49	.50	.41	.52	.55	.58	.60	1	.45	.53	.56	.47	.47	.58	.51	.45	.47	.47	.51	.50	.42	.49	.37	.43	.47	.53	.54	.33	.41	.58	.57	.59	.42
v10	.33	.33	.38	.29	.35	.31	.35	.37	.43	1	.37	.43	.37	.44	.42	.46	.29	.48	.47	.41	.34	.34	.39	.26	.31	.39	.36	.39	.24	.34	.36	.33	.35	.27
v11	.39	.40	.37	.33	.49	.43	.52	.49	.45	.35	1	.54	.51	.50	.58	.51	.47	.49	.45	.50	.45	.46	.48	.36	.40	.40	.45	.45	.30	.41	.46	.47	.44	.39
v12	.35	.39	.38	.38	.47	.48	.51	.48	.47	.29	.50	1	.60	.56	.59	.55	.53	.50	.49	.62	.56	.54	.59	.44	.50	.45	.50	.56	.35	.49	.50	.52	.52	.47
v13	.36	.33	.34	.30	.43	.42	.47	.42	.44	.34	.49	.52	1	.53	.57	.51	.51	.46	.43	.58	.55	.46	.51	.46	.51	.41	.45	.44	.30	.44	.42	.46	.46	.43
v14	.37	.41	.36	.37	.42	.40	.51	.46	.44	.31	.53	.52	.53	1	.62	.56	.52	.51	.46	.57	.53	.48	.53	.44	.56	.40	.42	.48	.38	.40	.44	.45	.50	.48
v15	.39	.42	.41	.32	.50	.47	.53	.50	.50	.34	.60	.53	.53	.60	1	.58	.59	.49	.49	.61	.57	.53	.61	.48	.56	.45	.54	.52	.36	.47	.53	.50	.56	.48
v16	.34	.37	.43	.34	.45	.40	.44	.43	.42	.33	.49	.46	.44	.51	.52	1	.47	.50	.49	.55	.45	.48	.51	.40	.51	.46	.43	.42	.30	.45	.41	.43	.44	.41
v17	.29	.36	.33	.29	.42	.38	.47	.40	.37	.22	.46	.49	.51	.49	.53	.41	1	.36	.34	.58	.60	.57	.59	.62	.58	.37	.50	.48	.38	.51	.42	.48	.52	.54
v18	.37	.36	.38	.30	.42	.40	.47	.40	.45	.41	.45	.42	.42	.43	.48	.45	.32	1	.61	.51	.43	.42	.46	.35	.38	.45	.39	.42	.24	.36	.42	.42	.41	.33
v19	.38	.35	.40	.35	.42	.41	.44	.45	.47	.42	.39	.40	.39	.39	.42	.44	.31	.45	1	.45	.41	.43	.45	.30	.39	.51	.42	.45	.29	.39	.42	.42	.42	.33
v20	.35	.37	.37	.37	.42	.41	.48	.45	.46	.28	.45	.56	.50	.51	.54	.50	.55	.42	.40	1	.60	.59	.60	.50	.51	.45	.51	.56	.39	.50	.51	.51	.51	.50
v21	.37	.42	.34	.39	.47	.48	.54	.48	.47	.30	.47	.55	.54	.55	.52	.44	.51	.40	.39	.57	1	.59	.55	.55	.49	.40	.53	.50	.43	.51	.49	.56	.56	.56
v22	.29	.33	.30	.26	.41	.42	.45	.41	.41	.26	.46	.46	.51	.49	.53	.41	.48	.37	.35	.49	.53	1	.56	.48	.50	.40	.49	.44	.40	.47	.46	.49	.48	.46
v23	.32	.40	.38	.32	.47	.44	.51	.48	.44	.29	.56	.52	.52	.59	.61	.51	.53	.43	.43	.56	.57	.54	1	.48	.57	.45	.49	.50	.36	.53	.51	.53	.50	.49
v24	.21	.30	.24	.21	.27	.30	.39	.31	.32	.18	.37	.42	.44	.44	.46	.36	.56	.28	.28	.47	.45	.45	.48	1	.53	.33	.41	.41	.41	.45	.38	.44	.42	.50
v25	.27	.31	.29	.22	.39	.35	.43	.40	.35	.24	.44	.44	.43	.46	.52	.50	.52	.35	.35	.46	.48	.50	.51	.49	1	.40	.42	.46	.43	.48	.42	.42	.48	.48
v26	.36	.33	.38	.35	.41	.41	.41	.40	.46	.38	.37	.44	.34	.36	.44	.42	.32	.45	.43	.38	.40	.36	.44	.28	.31	1	.48	.52	.29	.42	.45	.47	.50	.35
v27	.41	.45	.41	.41	.48	.47	.57	.53	.50	.30	.40	.44	.44	.50	.53	.40	.50	.37	.42	.52	.54	.42	.52	.42	.41	.45	1	.55	.39	.47	.59	.61	.60	.53
v28	.35	.37	.38	.36	.40	.39	.44	.43	.44	.30	.39	.43	.44	.43	.47	.40	.43	.37	.40	.45	.48	.42	.46	.36	.39	.46	.50	1	.35	.48	.52	.52	.58	.45
v29	.24	.31	.27	.29	.29	.35	.37	.33	.33	.19	.34	.33	.30	.36	.34	.30	.36	.25	.29	.34	.40	.29	.38	.26	.28	.29	.41	.34	1	.33	.37	.40	.41	.46
v30	.33	.34	.29	.29	.44	.40	.44	.42	.38	.22	.40	.39	.39	.39	.44	.36	.44	.33	.33	.39	.47	.37	.41	.36	.39	.37	.46	.39	.35	1	.45	.47	.48	.46
v31	.47	.47	.40	.38	.51	.57	.62	.69	.57	.35	.45	.42	.41	.46	.50	.40	.42	.41	.44	.45	.47	.43	.47	.32	.40	.45	.56	.48	.39	.43	1	.69	.63	.47
v32	.40	.44	.40	.35	.51	.59	.64	.62	.52	.30	.44	.50	.42	.45	.52	.39	.47	.38	.44	.52	.52	.46	.52	.43	.44	.43	.58	.45	.39	.46	.69	1	.67	.53
v33	.40	.43	.41	.39	.51	.51	.62	.57	.56	.32	.46	.48	.46	.50	.52	.42	.45	.39	.43	.49	.51	.48	.49	.42	.44	.45	.60	.47	.40	.45	.64	.66	1	.57
v34	.28	.32	.22	.25	.36	.37	.46	.42	.35	.20	.39	.44	.45	.47	.49	.36	.52	.30	.29	.41	.50	.52	.48	.49	.48	.32	.48	.39	.35	.41	.43	.52	.50	1

Note. All intracorrelations between items were significant at .01 alpha level. Correlations in upper and lower triangular matrixs were of male group (*N*=734) and female group (*N*=705), respectively.

Appendix D. Descriptive statistics of items by raw scores and indices scores for males (*N*=734) and females (*N*=705)

	Max	*Raw Mean*		*Raw SD*		*cp*		*mp*		*bp*		*wp*	
item	score	male	female	male	female	male	female	male	female	male	female	male	female
v1	2	1.30	1.39	0.93	0.90	.085	.074	.420	.445	.052	.052	.025	.020
v2	2	1.30	1.26	0.94	0.95	.084	.117	.416	.352	.056	.047	.026	.026
v3	3	1.85	2.05	1.40	1.33	.107	.085	.381	.420	.057	.052	.023	.020
v4	3	1.68	1.99	1.24	1.16	.141	.080	.308	.375	.065	.072	.035	.037
v5	2	1.27	1.29	0.95	0.94	.099	.113	.420	.385	.032	.029	.022	.021
v6	2	1.06	1.15	0.95	0.95	.188	.162	.282	.297	.040	.038	.029	.024
v7	3	1.65	1.76	1.38	1.34	.164	.150	.321	.326	.036	.027	.025	.022
v8	3	1.71	1.88	1.44	1.40	.148	.127	.357	.374	.031	.025	.016	.016
v9	3	1.93	2.11	1.34	1.26	.103	.080	.436	.469	.025	.028	.014	.013
v10	3	2.36	2.55	1.16	0.98	.028	.015	.632	.709	.027	.032	.009	.007
v11	2	1.23	1.23	0.97	0.96	.114	.130	.398	.351	.028	.025	.024	.028
v12	3	1.39	1.50	1.33	1.29	.265	.220	.214	.212	.034	.041	.028	.037
v13	4	1.74	1.90	1.71	1.68	.290	.231	.174	.195	.040	.040	.044	.045
v14	4	1.84	1.86	1.87	1.85	.263	.256	.215	.197	.034	.029	.029	.030
v15	3	1.47	1.57	1.49	1.48	.254	.212	.254	.266	.012	.016	.019	.018
v16	3	1.60	1.81	1.47	1.42	.185	.139	.296	.329	.035	.035	.024	.026
v17	4	1.03	1.04	1.64	1.61	.583	.559	.062	.048	.012	.009	.017	.022
v18	2	1.34	1.52	0.92	0.82	.080	.041	.463	.576	.034	.030	.017	.013
v19	2	1.32	1.50	0.81	0.74	.081	.048	.429	.520	.056	.045	.022	.020
v20	5	1.70	1.83	1.90	1.91	.426	.374	.103	.099	.032	.034	.033	.041
v21	3	0.95	1.18	1.33	1.39	.478	.359	.099	.133	.017	.020	.018	.024
v22	5	1.62	1.90	2.20	2.26	.465	.368	.098	.117	.019	.023	.029	.036
v23	5	1.94	2.28	2.34	2.42	.375	.276	.151	.199	.017	.018	.023	.020
v24	4	0.84	0.83	1.60	1.59	.681	.634	.037	.030	.006	.006	.018	.018
v25	3	0.93	0.94	1.38	1.36	.484	.462	.094	.076	.016	.016	.025	.032
v26	5	3.48	3.85	2.04	1.78	.060	.034	.497	.582	.039	.038	.019	.015
v27	3	1.22	1.45	1.40	1.43	.356	.242	.151	.217	.026	.027	.029	.025
v28	3	1.46	1.68	1.44	1.42	.244	.162	.234	.286	.032	.037	.029	.033
v29	3	0.54	0.80	1.10	1.26	.710	.521	.023	.040	.009	.022	.034	.046
v30	2	0.70	0.78	0.95	0.97	.402	.350	.122	.119	.026	.028	.032	.043
v31	3	1.40	1.68	1.43	1.39	.267	.174	.217	.291	.032	.036	.024	.018
v32	4	1.64	1.89	1.83	1.84	.349	.262	.158	.198	.026	.029	.027	.024
v33	3	1.24	1.40	1.36	1.32	.338	.259	.166	.191	.026	.035	.027	.027
v34	4	0.95	0.99	1.68	1.70	.630	.579	.051	.044	.008	.009	.017	.016
